# Nanopore sequencing of full-length *BRCA1* mRNA transcripts reveals co-occurrence of known exon skipping events

**DOI:** 10.1186/s13058-017-0919-1

**Published:** 2017-11-28

**Authors:** Lucy C. de Jong, Simone Cree, Vanessa Lattimore, George A. R. Wiggins, Amanda B. Spurdle, kConFab Investigators, Allison Miller, Martin A. Kennedy, Logan C. Walker

**Affiliations:** 1 Department of Pathology, University of Otago, Christchurch, New Zealand; 2 Genetics and Computational Biology Division, QIMR Berghofer Medical Research Institute, Queensland, Australia; 3 kConFab, Research Department, Peter MacCallum Cancer Centre, Melbourne, Australia; 4 The Sir Peter MacCallum Department of Oncology, University of Melbourne, Parkville, Australia

**Keywords:** *BRCA1*, Splicing, MinION, Nanopore sequencing, Full-length transcript, Exon skipping

## Abstract

**Background:**

Laboratory assays evaluating the effect of DNA sequence variants on *BRCA1* mRNA splicing may contribute to classification by providing molecular evidence. However, our knowledge of normal and aberrant *BRCA1* splicing events to date has been limited to data derived from assays targeting partial transcript sequences. This study explored the utility of nanopore sequencing to examine whole *BRCA1* mRNA transcripts and to provide accurate categorisation of in-frame and out-of-frame splicing events.

**Methods:**

The exon structure of *BRCA1* transcripts from a previously studied control lymphoblastoid cell line were assessed using MinION nanopore sequencing of long-range reverse transcriptase-PCR amplicons.

**Results:**

Our study identified and characterised 32 complete *BRCA1* isoforms, including 18 novel isoforms which showed skipping of multiple contiguous and/or non-contiguous exons. Furthermore, we show that known *BRCA1* exon skipping events, such as Δ(9,10) and Δ21, can co-occur in a single transcript, with some isoforms containing four or more alternative splice junctions. Fourteen novel isoforms were formed entirely from a combination of previously identified alternative splice junctions, suggesting that the total number of *BRCA1* isoforms might be lower than the number of splicing events reported previously.

**Conclusions:**

Our results highlight complexity in *BRCA1* transcript structure that has not been described previously. This finding has key implications for predicting the translation frame of splicing transcripts, important for interpreting the clinical significance of spliceogenic variants. Future research is warranted to quantitatively assess full-length *BRCA1* transcript levels, and to assess the application of nanopore sequencing for routine evaluation of potential spliceogenic variants.

**Electronic supplementary material:**

The online version of this article (doi:10.1186/s13058-017-0919-1) contains supplementary material, which is available to authorized users.

## Background

Routine diagnostic screening for deleterious variants in the breast cancer susceptibility gene *BRCA1* is typically performed for individuals from suspected high-risk breast (and ovarian) cancer families to identify the genetic cause for their disease. However, an important practical issue associated with genetic testing is the identification of rare sequence variants with unknown clinical significance. Interpreting the clinical meaning of unclassified variants is a key challenge facing the future of genomic-based health initiatives [1].

Multifactorial likelihood analysis is the most accepted approach for assessing cancer risk associated with unclassified *BRCA1* variants and has been successful in classifying hundreds of variants since it was developed [2, 3] (http://brcaexchange.org/). However, the multifactorial likelihood model is limited by the amount of information available from the variant carrier (tumour histopathology), the family of the variant carrier (co-segregation, family history information) and additional information, such as co-occurrence with a pathogenic variant. Numerous studies have shown that the effect of a variant of unknown clinical significance on *BRCA1* mRNA splicing may contribute to classification by offering molecular evidence [4–7]. Moreover, to classify variants using a combination of bioinformatic and in-vitro splicing data, Spurdle et al. [8] proposed five-tier splicing classification guidelines (Class 5, pathogenic; Class 4, likely pathogenic; Class 3, uncertain; Class 2, likely not pathogenic; Class 1, not pathogenic). These guidelines were subsequently improved after a multicentre study carried out by the international ENIGMA consortium [6].

Determining which mRNA splice isoforms are abnormal and potentially deleterious can be challenging. The ENIGMA Splicing Working Group recently undertook a comprehensive analysis to characterise numerous ‘naturally occurring’ mRNA splice isoforms for *BRCA1* to aid in the interpretation of in-vitro splicing assays [9]. This study identified more than 60 *BRCA1* mRNA isoform events occurring in breast and/or blood cells. However, it remains unclear whether these individual splicing events can co-occur in the same *BRCA1* transcript, as PCR-based and sequencing-based technologies used to assess splicing events typically interrogate only a fraction of the whole transcript(s). Pathogenic (or Class 5) variants that cause mRNA splicing changes are expected to disrupt protein function either through truncation or in-frame deletion of important regions of the encoded proteins. Using technologies that only examine a section of mRNA transcripts for variant classification may therefore lead to a misinterpretation of in-frame or out-of-frame splicing events.

DNA sequencing technology based on nanopore sequencing generates read lengths that greatly exceed those of more commonly used Sanger sequencing and massively parallel sequencing platforms. Moreover, nanopore sequencing has been demonstrated to characterise the complex exon structure of mRNA transcripts from genes expressing a large number of isoforms [10]. To our knowledge, single-molecule sequencing technologies (MinION [11] and PacBio [12]) that enable long sequence reads have yet to be employed to resolve the exon structure of whole *BRCA1* mRNA transcripts. In this study, we explored the utility of long-range reverse transcriptase (RT)-PCR with nanopore sequencing to identify novel *BRCA1* isoforms and the co-occurrence of known exon skipping events.

## Methods

### RNA sample

A human lymphoblastoid cell line (LCL) derived from a female healthy control, used in a previously reported study [7], was cultured with cycloheximide to prevent nonsense-mediated RNA decay (NMD), as described previously [5]. RNA was extracted from the cells using the RNeasy Mini Kit (Qiagen), according to the manufacturer’s instructions. The study participant provided written informed consent for research studies.

### cDNA synthesis

cDNA synthesis was carried out using oligo(dT) primers (ThermoFisher Scientific Inc.) and Superscript® III Reverse Transcriptase (ThermoFisher Scientific Inc.) according to the manufacturer’s instructions. Twenty microlitres of resulting cDNA mix was diluted 5-fold in H_2_O, and 3 μl of the final solution was used for each long-range PCR assay.

### PCR assays

Three different protocols were used to generate a pool of PCR amplicons for nanopore sequencing. PCR products were resolved in a 1% agarose gel using electrophoreses.

#### Protocol 1

Reactions contained 1.3 M betaine (Sigma-Aldrich), 1× KAPA long-range buffer (KAPA Biosystems), 1.75 mM MgCl_2_, 0.5 μM of each primer (BRCA1_1F 5′-GCGCGGGAATTACAGATAAA-3′ and BRCA1_24pR 5′-AAGCTCATTCTTGGGGTCCT-3′), 300 μM of KAPA, 200 μM dNTP mix, and 0.5 units of KAPA Long Range HotStart. Thermal cycling conditions were 94 °C for 4 minutes, followed by 35 cycles of 94 °C for 30 seconds, primer annealing at one of a range of temperatures (56.4–62.5 °C; Additional file 1: Figure S2) for 30 seconds, and 68 °C for 12 minutes, with a final extension of 72 °C for 12 minutes.

#### Protocol 2

PCR reactions contained 1 M betaine, 1× KAPA long-range buffer, 2 mM of MgCl_2_, 0.7 μM of each primer (BRCA1_1F and BRCA1_24pR), 200 μM dNTP mix, and 0.5 units of KAPA Long Range HotStart. Thermal cycling conditions were 94 °C for 2 minutes, followed by 35 cycles of 94 °C for 30 seconds, 55.7–62.5 °C (Additional file 1: Figure S2) for 30 seconds, and 68 °C for 7 minutes, before a final extension of 72 °C for 7 minutes.

#### Protocol 3

Reactions contained 1 M betaine, 1× KAPA long-range buffer, 2 mM MgCl_2_, 0.7 μM of each primer (BRCA1_1F and BRCA1_24pR), 200 μM dNTP mix, and 0.5 units of KAPA Long Range HotStart. Thermal cycling conditions were 94 °C for 2 minutes, then eight cycles of 94 °C for 30 seconds, 66 °C for 30 seconds (decreasing 1 °C each cycle), and 68 °C for 7 minutes, followed by 30 additional cycles of 94 °C for 30 seconds, 59 °C for 30 seconds, and 68 °C for 7 minutes, before a final extension of 72 °C for 7 minutes.

### Sanger sequencing

Sanger sequencing was carried out using Applied Biosystems Big Dye Terminator version 3.1 to confirm PCR products as described previously [13]. The Geneious® Multiple Sequence Aligner tool was used to match the Sanger sequence of the sample with the predicted isoform as a reference sequence.

### MinION library preparation, sequencing and alignment

The Oxford Nanopore MinION Genomic DNA Sequencing Kit (R9 flow cell chemistry) was used to prepare the DNA libraries according to the manufacturer’s instructions. Briefly, PCR products were purified and then quantified using the Qubit® Fluorometer (ThermoFisher Scientific) followed by end repair and dA tailing using the NEBNext Ultratm End Repair/dA-Tailing Module (New England BioLabs Inc.). The DNA library entailing adaptor ligation and purification of double-stranded DNA with hairpin adaptor was prepared using the Nanopore Sequencing Kit SQK-NSK007 (R9 version). The MinKNOW program was used for running MinION for 48 hours. Additional sample mix was applied to the flow cell when the number of pores being used was less than 20, until the entire sample was used. The raw electrical signal was uploaded to Metrichor (version 1.107), using the 2D Basecalling RNN for SQK-NSK007 which returned basecall data in MinION fast5 file format. The Poretools package was used to extract fasta files for high-quality 2D reads [14]. Sequence reads were mapped by the Genomic Mapping and Alignment Program (GMAP) [15] using the Linux command lines gmap -g [ReferenceSequence].fasta -f 2 -*n* 0 -t 16 [SequencesToAlign].fasta > [alignmentFile].gff3 as gmap -g BRCARD1_geneseq.fasta -f 2 -*n* 0 -t 16 all.fasta > all.gff3. The output file was manipulated using an in-house R script to select for reads spanning the full length of the gene by ensuring it contained sequences from the first and last exons. The full-length reads were then grouped into isoforms based on their composition of exons and introns.

## Results

### *PCR amplification of full-length* BRCA1 *cDNA*

**Fig. 1:**
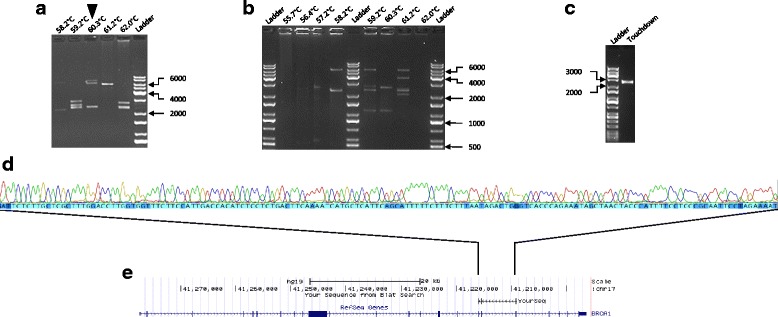
Example of fragments obtained from different long-range PCR assays from a LCL. Three different protocols were used to generate a pool of PCR amplicons for nanopore sequencing: **a** Protocol 1, **b** Protocol 2 and **c** Protocol 3. Primer annealing temperature indicated above each lane. Reference markers labelled for size in base pairs. **d**. Sanger sequence trace of PCR products in (**a**) (indicated by black triangle). **e**. BLAT alignment tool showing sequence match to *BRCA1* using the UCSC Genome Browser

Blood-based products such as lymphoblastoid cell lines (LCLs) have been widely used as a cell model for analysing *BRCA1* splicing changes in the clinical setting for variant evaluation [9]. For this study, we assessed RNA from a healthy control LCL that was used previously for an international workshop, led by the ENIGMA Consortium, comparing mRNA splicing assay protocols between laboratories [7]. To obtain full-length *BRCA1* transcripts, we carried out long-range RT-PCR for *BRCA1* transcripts using primers targeting the 3′ end of exon 1 and the 5′ end of exon 24 to generate a 5.8-kb amplicon (Additional file 1: Figure S1). Amplified products from repeat assays for a single LCL were visualised by gel electrophoresis, revealing a difference in patterns of amplicon sizes and suggesting variability in isoform selection and amplification during the PCR cycles (Fig. 1; Additional file 1: Figure S2). PCR products that were consistent by size with a full-length *BRCA1* isoform (NM_007294.3, encoding the full-length BRCA1 protein) were observed in 28/47 PCR assays. A preliminary assessment of fragments from 10 PCR assays by Sanger sequencing confirmed *BRCA1* identity (Fig. 1) prior to sequencing using MinION, suggesting a range of *BRCA1* isoforms were amplified. To maximise the number of whole *BRCA1* transcripts to be assessed by nanopore sequencing, amplified products were pooled from all 47 PCR assays using cDNA synthesised from a single LCL RNA sample.

### *BRCA1* isoform discovery and annotation

A total of 117,504 reads were obtained from MinION sequencing run from two DNA libraries (Library 1 = 105,482 reads; Library 2 = 12,022 reads) derived from pooled RT-PCR products on a R9 flow cell over a period of 48 hours. Approximately 21% of 2D reads containing both template and complement strand with a *Q*-value of 9 were obtained, of which 95% aligned to the target sequence. Reads failing the 2D filter were possibly due to shearing of DNA by pipetting or incomplete ligation of hairpin adaptors, thus resulting in shorter reads lacking a complementary strand signal. Our library contained pooled PCR products obtained from cDNA generated from *BRCA1* and *BARD1* (part of a separate study) transcripts. Nanopore sequencing resulted in 10.7% of passed 2D reads aligning to *BRCA1* and a higher proportion (84.4%) of passed 2D reads aligned to a shorter (≤2 kb) *BARD1* cDNA sequence. A summary of the mapping and filtering process is shown in Additional file 1: Figure S3. As a result of our stringent filtering criteria (2D reads that contain both exon 1 and exon 24 of *BRCA1*), a total of 177 reads were identified that revealed the complete structure of different *BRCA1* isoforms.

**Fig. 2:**
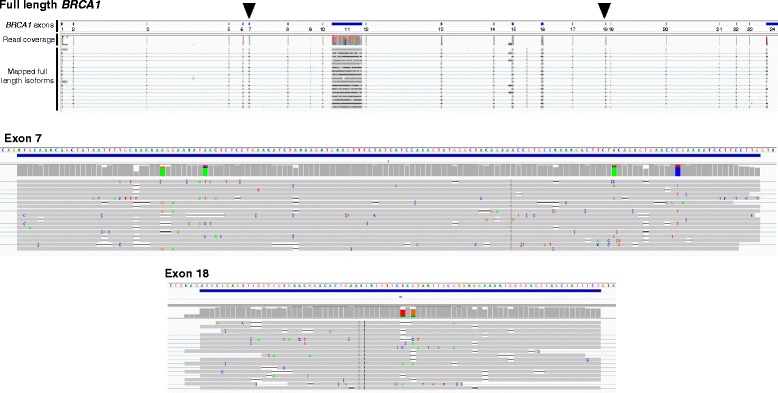
Sequencing of the full-length *BRCA1* transcript. Integrated Genome Viewer (IGV) screenshots for the whole gene, along with close-up views of exons 7 and 18 (also highlighted by black triangles on full-length *BRCA1*). *BRCA1* exons are indicated and represented as blue solid rectangles. Each MinION sequence read with perfect homology to the reference sequence shown in grey. Mismatches shown in colour and indicated by base

A total of 32 *BRCA1* isoforms (including full length; Fig. 2) were resolved with at least one sequencing read using a conservative mapping approach (GMAP) (Table 1). Of these, 20 isoforms have not been described previously. Of the 32 isoforms amplified by long-range RT-PCR, 23 lacked all or part of the largest *BRCA1* exon (exon 11; 3426 bases) (Table 1). Ten of these 23 isoforms contained a Δ11q splicing event rather than the complete skipping of exon 11. This result suggests that long-range RT-PCR assays were selective for shorter amplicons corresponding to smaller (<4 kb) isoforms and that the sequencing results from MinION may not be quantitative. Furthermore, the stringent quality control requirement for passed 2D reads would also eliminate DNA strands that may have been accidentally sheared by pipetting during library generation, thus potentially reducing quantitative measurement. The remaining nine of the detected transcripts were greater than 5 kb in length and included the full-length and Δ9,10 isoforms (Table 1), which have been shown previously to be ‘predominant’ transcripts in blood and breast cells using semi-quantitative measures [9]. It is therefore possible that amplicon selection during long-range RT-PCR cycles may also have been influenced by the relatively high proportion of transcript levels in a pool of *BRCA1* expressed isoforms.

**Table 1:** *BRCA1* isoforms identified by long-range PCR and nanopore sequencing

Isoform designation	RNA	Size of isoform (bp)	Number of MinION reads	In-frame/out-of-frame	Deleted protein domain(s)^a^	Previously described by Colombo et al. [9]^b^
Δ11q	r.788_4096del	3898	31	In-frame	–	Yes
Δ9,10,11q	r.548_670del + r.788_4096del	3775	27	In-frame	–	Yes
Full length	–	7207	17	In-frame	*–*	Yes
Δ10-17	r.594_5074del	2726	14	Out-of-frame	BCRT	No
Δ9,11q	r.548_593del + r.788_4096del	3852	10	Out-of-frame	–	Yes
11Δ3110	r.788_3897del	4093	8	In-frame	–	Yes
Δ1Aq,9,10,11q,12-15,16p	r.-25_-20del + r.548_670del + r.788_4804	3061	7	In-frame	–	No
Δ14	r.4358_4484del	7080	6	Out-of-frame	*–*	Yes
Δ10,11,17	r.594_ 4096del + r.4987_5074del	3616	5	In-frame	BCRT	**No**
Δ1Aq,11Δ3110,Δ14p,15-17	r.-25_-20del + r.788_3897del + r.4358_4360del + r.4485_ 5074del	3498	5	Out-of-frame	BCRT	**No**
Δ9,10	r.548_670del	7084	5	In-frame	*–*	Yes
Δ1Aq,5q,11q,14,21	r.-25_-20del + r.191_212del + r.788_4096del + r.4358_4484del + r.5278_5332del	3688	5	Out-of-frame	RING	**No**
Δ3	r.81_134del	7153	5	Out-of-frame	RING	Yes
Δ9,10,14	r.548_670del + r.4358_4484del	6957	3	Out-of-frame	*–*	**No**
Δ3-23	r.81_ 5467del	1820	3	Out-of-frame	RING, BCRT	No
Δ2,9,10,11q,▼21	r.-19_80del + r.548_670del + r.788_4096del + r.74421_74422ins74421 + 873_74421 + 1001	3676	3	Out-of-frame (loss of coding start site)	RING	No
Δ9-11	r.548_4096del	3658	3	In-frame	–	Yes
Δ2,9,10,11Δ3110,Δ14,20,22	r.-19_80del + r.548_670del + r.788_3897del + r.4358_4484del + r.5194_5277del + r.5333_5406del	3586	3	Out-of-frame (loss of coding start site)	RING, BCRT	**No**
Δ11q,21	r.788_4096del + r.5278_5332del	3843	3	Out-of-frame	BCRT	**No**
Δ11	r.671_4096del	3781	2	In-frame	–	Yes
Δ3,11Δ3110	r.81_134del + r.788_3897del	4039	1	Out-of-frame	RING	**No**
Δ9,10,21	r.548_670del + r.5278_5332del	7029	1	Out-of-frame	BCRT	**No**
Δ21	r.5278_5332del	7152	1	Out-of-frame	BCRT	Yes
Δ3,9,10,11q	r.81_134del + r.548_670del + r.788_4096del	3721	1	Out-of-frame	RING	**No**
Δ15-17	r.4485_ 5074del	6617	1	Out-of-frame	BCRT	Yes
11Δ3110,14,20-22	r.788_3897del + r.4358_4484del + r.5194_ 5406del	3753	1	Out-of-frame	BCRT	No
Δ8,11	r.442_547 + r.671_4096del	3675	1	Out-of-frame	–	No
11Δ3110,Δ14	r.788_3897del + r.4358_4484del	3966	1	Out-of-frame	–	**No**
Δ11q,19,20	r.788_4096del + r.5153_5193_ + r.5194_5277del	3773	1	Out-of-frame	BCRT	No
Δ5,9,10,11q	r.135 _212del + r.548_670del + r.788_4096del	3697	1	In-frame	RING	**No**
Δ3,9	r.81_134del + r.548_593del	7107	1	Out-of-frame	RING	**No**
11Δ3110,Δ21,23	r.788_3897del + r.5278_5332del + r.5407_5467del	3977	1	Out-of-frame	BCRT	**No**

*bp* base pairs, *BRCT* BRCA1 C-terminal domain, *RING* RING finger domain, Δ whole or partial exon skipping, ▼ partial intron retention

^a^Only clinically important BRCA1 protein domains are shown (ENIGMA BRCA1/2 Gene Variant Classification Criteria, Version 2.5 29 June 2017, https://enigmaconsortium.org/library/general-documents/). Position of amino acid residues denoting BRCA1 protein domains are as follows: RING, 1–101 and BCRT, 1651–1863

^b^Novel isoforms that are created entirely from previously identified partial or whole exon skipping events are shown in bold

Colombo et al. [9] previously characterised a total of 63 *BRCA1* alternative splicing events, 17 of which were detected and further validated in this study. Eighteen of the 20 full-length novel isoforms identified by MinION sequencing were found to contain co-occurring exon skipping events (Table 1). Many of these events found to co-occur, such as Δ(9,10), Δ11q, Δ(9,10,11q) and Δ3, have been identified previously in isolation using partial transcript analyses by Colombo et al*.* [9]. Of the 20 novel isoforms discovered in this study, 14 were formed entirely from a combination of previously identified alternative splice junctions (Table 1). While these data show that many non-contiguous exon skipping events for *BRCA1* mRNA occur concurrently, this analysis was possibly non-quantitative and therefore was unable to establish the relative levels of different transcripts.

Two novel isoforms identified, Δ10-17 (Additional file 1: Figure S4) and Δ3-23, show skipping of multiple contiguous exons generating out-of-frame coding sequences. We are unaware of previous studies that have implemented a PCR-based assay design that has encompassed exons 10–17 or exons 3–23. It is therefore not surprising that a long-range PCR-based approach has for the first time detected such isoforms. It is unlikely that Δ10-17 and Δ3-23 give rise to functional proteins as they lack the BRCA1 C-terminal (BCRT) domain (Table 1). Furthermore, the out-of-frame coding sequences for these isoforms suggest that they would be susceptible to NMD [16].

**Fig. 3:**
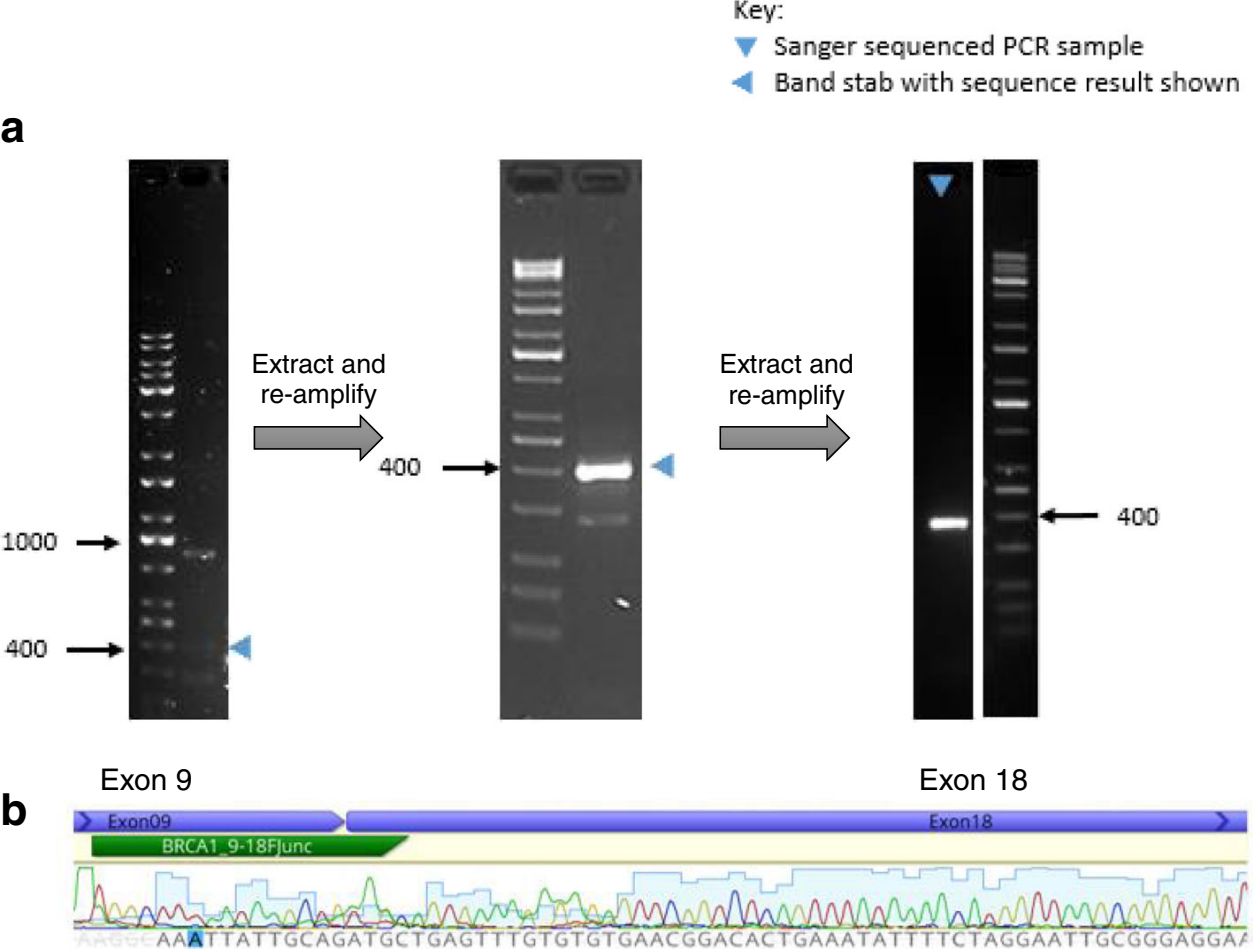
RT-PCR and Sanger sequencing confirmation of isoform ∆10-17. **a** RT-PCR analysis of mRNA isolated from a cycloheximide-treated LCL. To obtain a single isoform amplicon with sufficient DNA for sequence analysis, band extraction (shown by blue arrowheads) and re-amplification were carried out twice. The final 408-bp product was analysed by Sanger sequencing. Reference markers labelled for size in base pairs. **b** Sanger sequence trace of the exon 9–18 novel splice junction from the PCR product indicated in (**a**). Location of BRCA1_9-18 junction specific PCR primer (BRCA1_9-18FJunc) indicated in green

The novel Δ10-17 isoform was selected for validation by Sanger sequencing as the isoform was common within the PCR amplicon library, and had a single junction which was relatively straightforward to amplify. RT-PCR assays using oligonucleotide primers targeting the exon 9–18 junction, followed by Sanger sequencing, confirmed the presence of this novel isoform (Fig. 3).

Together, these results suggest complexity in transcript structure that has not been described previously for *BRCA1*. Because of the potential error rate of MinION (>10%) [17], a higher read depth would increase the confidence in 19 of the 32 characterised transcripts represented by a relatively small number (*n* ≤ 3) of reads, and this would be particularly important for potential splice shift events not identified previously.

### Co-occurring splicing events and interpretation for variant classification

Determining whether *BRCA1* transcripts lead to abnormal and potentially deleterious proteins requires knowledge about the structure of the coding isoforms. Sixteen of the 20 novel isoforms lacked sequences coding for the RING and/or BCRT domains, which have been previously shown to harbour amino acid residues of clinical importance [18], although 14 of these 16 isoforms are out-of-frame and would therefore be susceptible to NMD. The remaining two isoforms, Δ(10,11,17) and Δ(5,9,10,11q), are predicted to be in-frame and may potentially give rise to proteins lacking the clinically significant BCRT and RING domains, respectively.

We detected the predominant splicing events, Δ11q and Δ9,10, which individually would be predicted to generate modified in-frame transcripts that are not considered deleterious based on protein coding. However, our study has shown these splicing events can co-occur with skipping and insertion events causing out-of-frame coding in these isoforms (Table 1). Two examples were the Δ(9,10,21) and Δ(11q,21) isoforms which cause out-of-frame coding due to the exon 21 skipping event. Furthermore, the combination of multiple out-of-frame exon skipping events can result in segments of the resulting transcript being in-frame. For example, Δ10,11 in the Δ(10,11,17) isoform is an out-of-frame deletion, but together with the out-of-frame Δ17 event the isoform returns to being in-frame coding from exon 18 to exon 24. This transcript lacks sequence for the BRCT domain, suggesting that the isoform may avoid NMD and generate a protein that does not retain full BRCA1 function. Obtaining whole transcript information may therefore have important implications for interpreting the biological and clinical significance of spliceogenic variants.

## Discussion

Laboratory assays assessing the effect of DNA sequence variants on *BRCA1* mRNA splicing may contribute to classification by offering molecular evidence [5, 6]. However, detection of *BRCA1* splicing events to date has been restricted to assays targeting segments within *BRCA1* transcripts [9]. For the first time, we show that MinION nanopore sequencing of long-range PCR amplicons is able to resolve the exon structure of whole *BRCA1* transcripts, enabling accurate prediction of in-frame and out-of-frame coding events. Our study identified 20 novel *BRCA1* isoforms, 18 of which contained multiple individual splicing events. Many of the individual *BRCA1* exon skipping events and splice donor shifts (e.g. Δ1Aq, Δ5q and 11q) have been found previously [9]; however, our data indicate that these events can co-occur within single transcripts. Such complexity in transcript structure has not been described previously for *BRCA1* and has potential implications for interpreting the biological and clinical significance of spliceogenic variants.

While our work highlights that many *BRCA1* mRNA splicing events occur concurrently, our non-quantitative study was unable to establish the likelihood of such events being expressed in the same transcript. If future studies show that many of the detected *BRCA1* exon skipping events exclusively co-occur, then the total number of isoforms might be lower than the number of splicing events reported previously by Colombo et al. [9]. Such a finding would suggest similar splicing patterns may also exist for genes other than *BRCA1*. Further studies will therefore be required to measure the cellular levels of sequenced isoforms, and to investigate the possibility of further transcript complexity due to splice site shifts involving a small number of nucleotides. Such studies will require improved data analysis tools to take full advantage of the sequencing information generated, although we note that this field continues to advance as evidenced in a recent report by Hu et al. [19].

The ENIGMA Splicing Working Group previously led a multicentre study which highlighted methodological issues that confounded the interpretation of splicing results [6]. A major reason for these issues was determined to be PCR assay design and the restrictive positioning of primers which prevented detection of additional naturally occurring isoforms. Our follow-up study has been successful in demonstrating the capability of the MinION device to characterise the exon structure of whole *BRCA1* transcripts. Together, our results highlight the potential of this technology to overcome limitations of traditional PCR-based techniques.

## Conclusions

Our study highlights complexity in *BRCA1* transcript structure that has not been described by previously reported studies. Assessment of whole *BRCA1* transcripts is now possible and has key implications for predicting translation frame of splicing transcripts, which is important for interpreting the clinical significance of spliceogenic variants. Future research is warranted to quantitatively assess full-length *BRCA1* transcript levels, to detect additional novel isoforms involving small nucleotide shifts and to assess the application of nanopore sequencing for routine evaluation of potential spliceogenic variants. Furthermore, the application of MinION or similar platforms may be extended to other disease-associated genes to establish whether they display similar complex splicing patterns to *BRCA1*.

## Additional file

Additional file 1: is **Figure S1** Showing primer design to span *BRCA1* cDNA. Primers indicated by arrows positioned on exon 1 and exon 24. Full-length transcript is 7.2 kb. Length of full length amplicon is 5.8 kb. **Figure S2** showing results from 47 *BRCA1* RT-PCR assays. 2 μl of each reaction was visualised on a 1% agarose gel. Samples that underwent MinION sequencing in Sample 1 or Sample 2 are indicated, and long-range PCR reactions where we confirmed fragments were specific to *BRCA1* by Sanger sequencing. 2 μl of each reaction was run on 1% agarose gels. **A** Protocol 1. **B** Protocol 2. **C** Protocol 3. Primer annealing temperature indicated above each lane. Reference markers labelled for size in base pairs. **Figure S3** showing a schematic of the nanopore sequencing read filtering steps applied in the study. **Figure S4** showing sequencing of the full-length *BRCA1* Δ10-17 isoform. Integrated Genome Viewer (IGV) screenshots shown for the whole gene, along with close-up views of exons 9 and 18 (also highlighted by black triangles). *BRCA1* exons are indicated and represented as blue solid rectangles. Each MinION sequence read with perfect homology to the reference sequence is shown in grey. Mismatches are shown in colour and indicated by base. (DOCX 3674 kb)

## Abbreviations

BCRT: BRCA1 C-terminal domain
BLAT: BLAST-like alignment tool
cDNA: Complementary DNA
ENIGMA: Evidence-based Network for the Interpretation of Germline Mutant Alleles
GMAP: Genomic Mapping and Alignment Program
LCL: Lymphoblastoid cell line
NMD: Nonsense-mediated decay
PCR: Polymerase chain reaction
RT: Reverse transcriptase
