# Supplementary material for: Nanopore sequencing of full-length BRCA1 mRNA transcripts reveals co-occurrence of known exon skipping events
Source: Breast Cancer Res. 2017 Nov 28;19:127. doi: 10.1186/s13058-017-0919-1 (PMC5706335; doi:10.1186/s13058-017-0919-1)
Supplement: Additional file 1: — is Figure S1 Showing primer design to span BRCA1 cDNA. Primers indicated by arrows positioned on exon 1 and exon 24. Full-length transcript is 7.2 kb. Length of full length amplicon is 5.8 kb. Figure S2 showing results from 47 BRCA1 RT-PCR assays. 2 μl of each reaction was visualised on a 1% agarose gel. Samples that underwent MinION sequencing in Sample 1 or Sample 2 are indicated, and long-range PCR reactions where we confirmed fragments were specific to BRCA1 by Sanger sequencing. 2 μl of each reaction was run on 1% agarose gels. A Protocol 1. B Protocol 2. C Protocol 3. Primer annealing temperature indicated above each lane. Reference markers labelled for size in base pairs. Figure S3 showing a schematic of the nanopore sequencing read filtering steps applied in the study. Figure S4 showing sequencing of the full-length BRCA1 Δ10-17 isoform. Integrated Genome Viewer (IGV) screenshots shown for the whole gene, along with close-up views of exons 9 and 18 (also highlighted by black triangles). BRCA1 exons are indicated and represented as blue solid rectangles. Each MinION sequence read with perfect homology to the reference sequence is shown in grey. Mismatches are shown in colour and indicated by base. (DOCX 3674 kb) [file 13058_2017_919_MOESM1_ESM.docx]

**Additional file 1**

BRCA1_24pR

Exon 1

Exon 24

5.8kb

7.2kb

BRCA1_1F

**Figure S1.** Primer design to span BRCA1 cDNA. Primers indicated by arrows positioned on Exon 1 and Exon 24. Full length transcript is 7.2kb. Length of full length amplicon is 5.8kb.

**Figure S2.** Results from 47 BRCA1 RT-PCR assays. 2μl of each reaction was visualised on a 1% agarose gel. Samples that underwent MinION sequencing in Sample 1 or Sample 2 are indicated, and long range PCR reactions where we confirmed fragments were specific to BRCA1 by Sanger sequencing. 2μl of each reaction was run on 1% agarose gels. A: Protocol 1. B: Protocol 2. C: Protocol 3. Primer annealing temperature is indicated above each lane. Reference markers labelled for size in base pairs.

**Figure S3.** Schematic of the nanopore sequencing read filtering steps applied in the study.

**Figure S4.** Sequencing of the full-length *BRCA1* Δ10-17 isoform. Integrated Genome Viewer (IGV) screenshots are shown for the whole gene, along with close-up views of exons 9 and 18 (also highlighted by black triangles). *BRCA1* exons are indicated and represented as blue solid rectangles. Each MinION sequence read with perfect homology to the reference sequence is shown in grey. Mismatches are shown in colour and indicated by base.
